# Analysis and Application of Drought Characteristics Based on Theory of Runs and Copulas in Yunnan, Southwest China

**DOI:** 10.3390/ijerph17134654

**Published:** 2020-06-28

**Authors:** Liping Wang, Xingnan Zhang, Shufang Wang, Mohamed Khaled Salahou, Yuanhao Fang

**Affiliations:** 1 College of Hydrology and Water Resources, Hohai University, Nanjing 210098, China; lpwang2009@hotmail.com (L.W.); yuanhao.fang@outlook.com (Y.F.); 2 College of Water Conservancy, Yunnan Agricultural University, Kunming 650201, China; sfwang@ynau.edu.cn; 3 State Key Laboratory of Hydrology–Water Resources and Hydraulic Engineering, Hohai University, Nanjing 210098, China; salahou@hhu.edu.cn

**Keywords:** drought, theory of runs, copula, joint return period, Yunnan province

## Abstract

Drought is a complex natural disaster phenomenon. It is of great significance to analyze the occurrence and development of drought events for drought prevention. In this study, two drought characteristic variables (the drought duration and severity) were extracted by using the Theory of Runs based on four drought indexes (i.e., the percentage of precipitation anomaly, the standardized precipitation index, the standardized precipitation evapotranspiration index and the improved comprehensive meteorological drought index). The joint distribution model of drought characteristic variables was built based on four types of Archimedean copulas. The joint cumulative probability and the joint return period of drought events were analyzed and the relationship between the drought characteristics and the actual crop drought reduction area was also studied. The results showed that: (1) The area of the slight drought and the extreme drought were both the zonal increasing distribution from northeast to southwest in Yunnan Province from 1960 to 2015. The area of the high frequency middle drought was mainly distributed in Huize and Zhanyi in Northeast Yunnan, Kunming in Central Yunnan and some areas of Southwest Yunnan, whereas the severe drought was mainly occurred in Deqin, Gongshan and Zhongdian in Northwest Yunnan; (2) The drought duration and severity were fitted the Weibull and Gamma distribution, respectively and the Frank copula function was the optimal joint distribution function. The Drought events were mostly short duration and high severity, long duration and low severity and short duration and low severity. The joint cumulative probability and joint return period were increased with the increase of drought duration and severity; (3) The error range between the theoretical return period and the actual was 0.1–0.4 a. The year of the agricultural disaster can be accurately reflected by the combined return period in Yunnan Province. The research can provide guidelines for the agricultural management in the drought area.

## 1. Introduction

Drought is a natural phenomenon of water shortage in the process of water circulation [1]. The process of drought occurrence is complex, progressive and has a widespread influence. It will lead to crop yield loss, food shortage and environment degradation, which can cause great destruction to the economic and life [2,3,4]. The global surface temperature has increased about 0.85 degrees from 1880–2012, and the future temperature would continue to rise in the fifth assessment report of the Intergovernmental Panel on Climate Change (IPCC) [1]. The frequency of the extreme drought events happened will be higher under the global climate and environmental changes. The drought duration, severity and impact range in 2012–2015 had reached the highest in the same period in history in California, USA, where the return period of drought was close to 21,000 a [5]. However, a successive drought in autumn, winter and spring was occurred in the southwest China in 2009–2010. Therefore, analyzing the characteristics of regional drought events was especially important and will help the government to make the preventive measure to the drought.

Drought index is essential in drought research. Selecting appropriate drought index, identifying the drought process, and extracting the characteristic variables of drought events are the key points in drought characteristic analysis. To date, only one type of the drought indexes was used to analyze the drought characteristics in the previous studies [6,7,8,9,10,11]. However, only one type of drought index cannot accurately describe the spatial–temporal characteristics of drought in different areas. The drought index should be chosen effectively and reasonably according to the actual situation because the applicability of drought indexes is not the same in different regions. In the study, the percentage of precipitation anomaly (Pa), the standardized precipitation index (SPI), the standardized precipitation evapotranspiration index (SPEI) and the improved comprehensive meteorological drought index (CI_new_) were used to extract the drought characteristic variables, according to the conclusion of the applicability of the drought index in our previous study [12].

The Theory of Runs is a time series analysis method, which has been widely used in the identification of drought process [13,14,15]. Its basic problem is the determination of the intercept level. In previous research, many researchers only set a single intercept level in the Theory of Runs [11,16,17]. This method was simple, but it was tended to over identify or incomplete identify drought events, which affected the accuracy of the results. Therefore, it is necessary to further optimize the interception level of drought identification, and test more different drought index thresholds, so that the drought events identified can be consistent with the actual ones.

The theoretical basis of Copula function is Sklar’s theorem [18], which is an effective method to describe the dependence between variables without requiring the same type of edge distribution of each single variable when constructing the joint distribution. It is widely used in meteorological drought [19], hydrological drought [8], flood disaster [20] and other study fields. Tosunoglu [21] chose the annual maximum drought severity and the corresponding duration as the characteristic variables and used Gumbel–Hougaard Copula function to analyze the drought characteristics of Turkey. Kwon [22] calculated the return period of extreme drought events in 2013–2015 on the Korean Peninsula, based on Copula function of Bayesian framework. Throughout the existing research, Copula function was rarely used in agricultural disaster related fields until now. There are significant regional changes in drought characteristics for the complexity of drought and the difference of climate change.

There is mainly the dry farming in the plateau in Yunnan Province, where the terrain and landform are complex, the regional differences and vertical changes of climate are obvious the drought disasters occurred frequently. In 2009–2013, the cumulative disaster area of crops was about 4.9 million km^2^ for four consecutive years and the direct economic loss of 39.6 billion yuan [23]. Some researchers had analyzed the temporal and spatial evolution characteristics and the laws of drought in Yunnan Province [24,25,26]. However, most of these studies were based on single variable, single drought index or analyzed the reasons of some specific drought years. Currently, there is no study on the characteristics of drought probability and return period based on the combining the Theory of Runs and the copula function and the combining the agricultural drought and meteorological drought.

Therefore, the objectives of this study are to: (1) Separate the two characteristic variables (the drought duration and severity) from the drought index series by using the Theory of Runs, based on the meteorological data on 29 meteorological stations in Yunnan Province from January 1960 to December 2015; (2) Use the copula function to establish the joint distribution function of the two variables to analyze the joint cumulative probability and the joint return period of the drought events and (3) Compare the drought characteristics with the actual agricultural drought disaster in Yunnan Province from 1991 to 2015 to text the reliability of the method, so as to provide guidelines for the agricultural management in the drought area.

## 2. Study Area and Data

**Figure 1:**
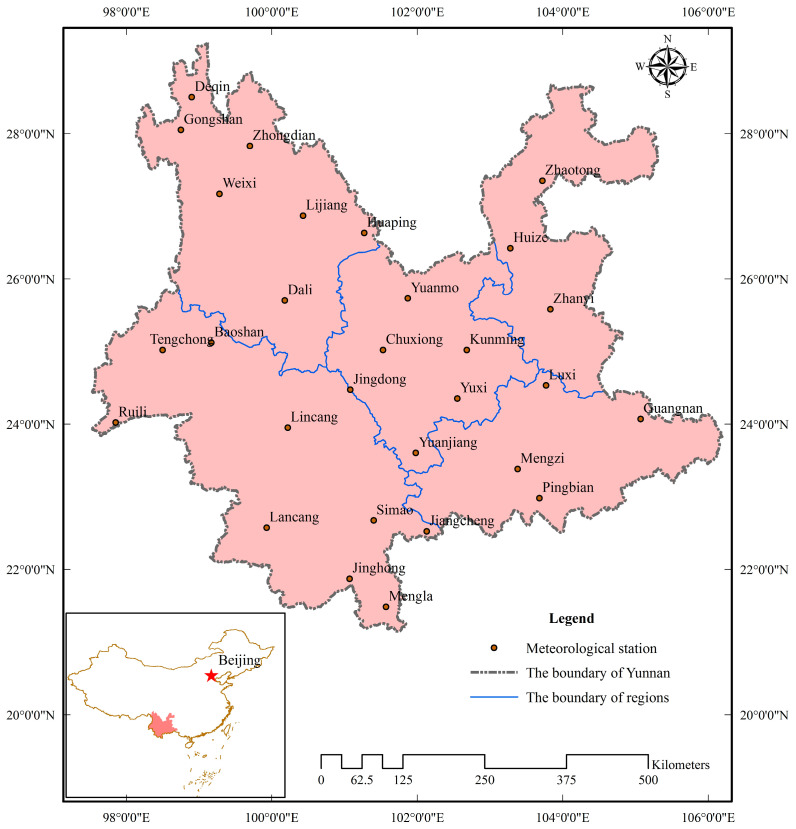
The location of the meteorological stations in Yunnan Province.

Yunnan Province is located in the southwest of China, the latitude is 20°8′32″–29°15′8″ N and the longitude is 97°31′39″–106°11′47″ E. The total area is 3.94 × 10^5^ km^2^. Because of the complex topography and special geographical location, the characteristics of the climate include latitude climate, monsoon climate and mountain climate. The average annual precipitation is 1110 mm, and the annual and regional distribution is very uneven, especially the precipitation in the dry season was only about 15% of the whole year. The drought events occurred frequently [27,28,29]. For example, a severe and sustained drought from autumn 2009 until spring 2010, resulted in the drying up of 744 streams, 564 small reservoirs and 7599 ponds [30,31]. Figure 1 shows the location of the meteorological stations in Yunnan Province.

Meteorological data comes from the China meteorological data sharing service system (http://data.cma.cn). The monthly data sets of 29 meteorological stations in Yunnan Province from January 1960 to December 2015 were selected mainly considering the integrity, consistency and reliability of the data series and the spatial representativeness of the stations. The data of agricultural drought disaster comes from Yunnan statistical yearbook, China Meteorological Disaster code (Yunnan volume), Yunnan water drought disaster, the China meteorological data sharing service system (http://data.cma.cn) and the China Planting Information Network (http://www.zzys.moa.gov.cn).

## 3. Methodology

### 3.1. Drought Index

The applicability evaluation of each drought index in different regions of Yunnan Province was obtained from the previous study (Table 1) [12]. In the winter and spring, SPI was most suitable in Northwest and Northeast Yunnan, SPI and Pa were recommended in Central Yunnan, Pa was more applicable in Southwest Yunnan and CI_new_ had better monitoring effect in Southeast Yunnan. In the summer and autumn, the SPEI was more applicable in the areas except the SPI was more suitable in Northwest Yunnan. The calculation of each drought index can be found in the previous research [12]. According to the standard classification of meteorological drought [32], drought events can be divided into five grades based on SPI, SPEI, Pa and CI_new_ values showed in Table 2. More severe grades of drought have smaller values of SPI, SPEI, Pa and CI_new_. No drought is defined as SPI / SPEI > −0.5, Pa > −0.4, CI_new_ > −0.6; the slight drought is defined as SPI / SPEI between −1.0 and −0.5, Pa between −0.6 and −0.4, CI_new_ between −1.2 and −0.6; the moderate drought is defined as SPI / SPEI between −1.5 and −1.0, Pa between −0.8 and −0.6, CI_new_ between −1.8 and −1.2, the severe drought is defined as SPI / SPEI between −2.0 and −1.5, Pa between −0.95 and −0.8, CI_new_ between −2.4 and −1.8, and the extreme drought is defined as SPI / SPEI ≤ −2.0, Pa ≤ −0.95, CI_new_ ≤ −2.4.

### 3.2. Drought Event Identification

**Figure 2:**
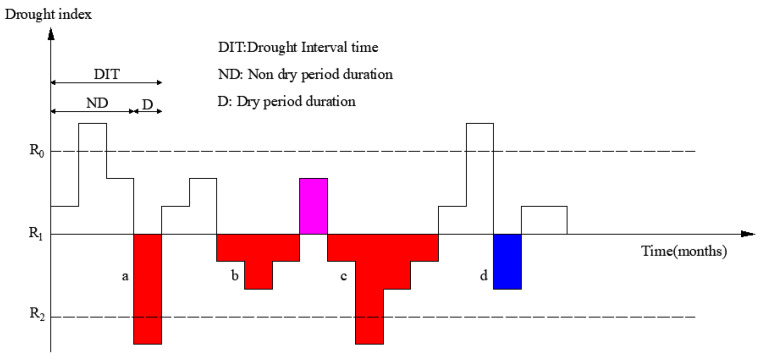
Definition of drought events based on drought index using Theory of Runs.

Yevjevich [33] proposed the Theory of Runs as a tool to define drought and study its properties. “Run” means a series of the same symbol that satisfies certain condition. Run length is the number of the same symbols in a run. This theory is based on the selection of a proper threshold. That is, according to the relationship between the drought index value and the threshold, to identify the beginning, continuation or end of drought. In this study, drought duration and severity were separated from the drought index series as the drought characteristics factors by using the Theory of Runs [34,35,36]. Drought duration is the duration from the beginning to the end of the drought event, and drought severity is the cumulative sum of the difference between the drought index value and its threshold value in the drought event process. Three different drought index thresholds (i.e., R_0_, R_1_ and R_2_) were set in this study (Figure 2). R_0_ is mainly used to combine the drought events, R_1_ is mainly used to identify drought events with low drought severity, R_2_ is mainly used to identify drought events with high drought severity. The values of R_0_, R_1_ and R_2_ is determined by the trial-and-error method. When determining the threshold value of the drought index, the results of the drought event identification should be consistent with the actual drought events recorded in the documentation [37].

The duration of drought is the duration from the beginning to the end of the drought event, and the drought intensity is the cumulative sum of the difference between the drought index value and its threshold value in the drought process.

In this study, one month is used as a time unit for the drought event identification. The method is as follows [38]:

(1) Preliminary identification. When the drought index value is less than R_1_, the month is determined initially as drought (i.e., the events of a, b, c and d in Figure 2).

(2) Deleting the non-drought events. For the drought events that lasted only one month (e.g., the events of a and d), if the drought index value is less than R_2_ (e.g., the event of a), the month will be considered a drought event, otherwise, the month will not be considered a drought event (e.g., the event of a) and will be deleted.

(3) Combined the drought events. The two drought events will be combined into one drought event (e.g., the events of b and c are combined into one drought event), when the time interval between the two adjacent drought events is only one time unit, and the drought index value of the interval period is less than R_0_. Otherwise, the two drought events will be considered as two independent drought processes.

From Figure 2, the drought duration combined is:

(1) $$D=d_{b}+d_{c}+1$$

where *D* is the combined drought duration; $d_{b}$ and $d_{c}$ are the drought duration of the events of b and c, respectively.

The drought severity combined is:

(2) $$S=s_{b}+s_{c}$$

where *S* is the combined drought severity; $s_{b}$ and $s_{c}$ are the drought severity of the events of b and c, respectively. 

### 3.3. Marginal Distribution of Characteristic Variables

Six single variable distribution functions (i.e., Gamma distribution, Exponential distribution, Log-normal distribution, Poisson distribution, Generalized Extreme Value distribution and Weibull distribution) were used to fit and optimize the marginal distribution of the two characteristic variables (i.e., drought duration and drought severity). Kolmogorov Smirnov (K–S) method was used to test the theoretical distribution in order to determine the optimal marginal distribution function, and the maximum likelihood estimation method was used to estimate the parameters. The principle of the calculation can be found in the literature [39]. Kendall′s rank correlation coefficient [40] was used to test the correlation between the characteristic variables.

### 3.4. The Joint Distribution Model Based on Two-Dimensional Copulas

Copulas are functions introduced by Sklar [18] for linking univariate distribution functions to multivariate distribution functions. The merit of using copulas is that marginal distribution of each variable is unlimited, and the variables can be correlated, it has good flexibility and adaptability. For two-dimensional copulas, if the marginal distribution function of the drought duration and severity are *F_D_*(*d*) and *F_S_*(*s*), respectively, the joint distribution function of the two-dimensional copulas is as follows:

(3) $$F\left(d,s\right)=P\left(D\leq d,S\leq s\right)=C\left(F_{D}\left(d\right),F_{S}\left(s\right)\right)=C\left(u,v\right)$$

where *D* and *S* is the combined drought duration and combined drought severity, respectively; *d* and *s* are the drought duration and severity, respectively. *C* is the copula function; *u* and *v* are the univariate cumulative distribution functions.

There are three common types of copula joint functions: Archimedean, Elliptic–Meta and Copula Quadratic. Archimedean copulas have advantages of simple form, symmetrical structure, strong combination, which other copulas do not have. Four types of Archimedean copulas have been widely used in the area of hydrology and, in particular, in the analysis of characteristics of drought [41,42,43,44]. In this study, the Archimedean copulas (Table 3), including Gumbel–Hougaard (G–H), Ali–Mikhail–Haq (A–M–H), Clayton and Frank copulas, were selected to analyze the joint probability of drought events. G–H copula can capture the characteristics of the upper tail on the variable frequency curve but is not sensitive to the change of the bottom tail. Contrary to G–H copula, Clayton copula is sensitive to the change of the bottom tail. Frank copula cannot capture the asymmetric relationship like G–H and Clayton copula because of its symmetry. G–H and Clayton copula are suitable for the case of positive correlation between variables, so they are suitable for the joint distribution of positive correlation between variables such as drought duration and severity. Frank and (A–M–H) can combine both positive and negative correlation variables, which has no limitation on correlation. Root Mean Square Error (RMSE) and Akaike information criteria (AIC) are used to test the fitting. The smaller the values of the RMSE and AIC, the better the fitting [39]. The parameter (θ) of the copula can be calculated by Kendall’s correlation coefficient between the drought duration and severity [45].

### 3.5. Return Period of Drought

According to the theory of return period [46] the formula for the return period of the drought duration (*D*) and the drought severity (*S*) are as follows:

(4) $$T_{D}=\frac{E\left(L\right)}{1-F_{D}\left(d\right)}$$

(5) $$T_{S}=\frac{E\left(L\right)}{1-F_{S}\left(s\right)}$$

where *T_D_* is the return period for drought duration and Ts is the return period for drought severity; *E*(*L*) is the expectation of drought interval, which is the sum of the average values of the drought duration and non-drought duration. The joint return period of drought duration and severity is defined as follows [47]:

(6) $$T_{DS}^{’}=\frac{E\left(L\right)}{P\left(D\geq dorS\geq s\right)}=\frac{E\left(L\right)}{1-C\left(F_{D}\left(d\right),F_{S}\left(s\right)\right)}$$

where $T_{DS}^{’}$ is the return period for D≥d or S≥s.

## 4. Results

### 4.1. Spatial Distribution Characteristics of Drought Frequency

**Figure 3:**
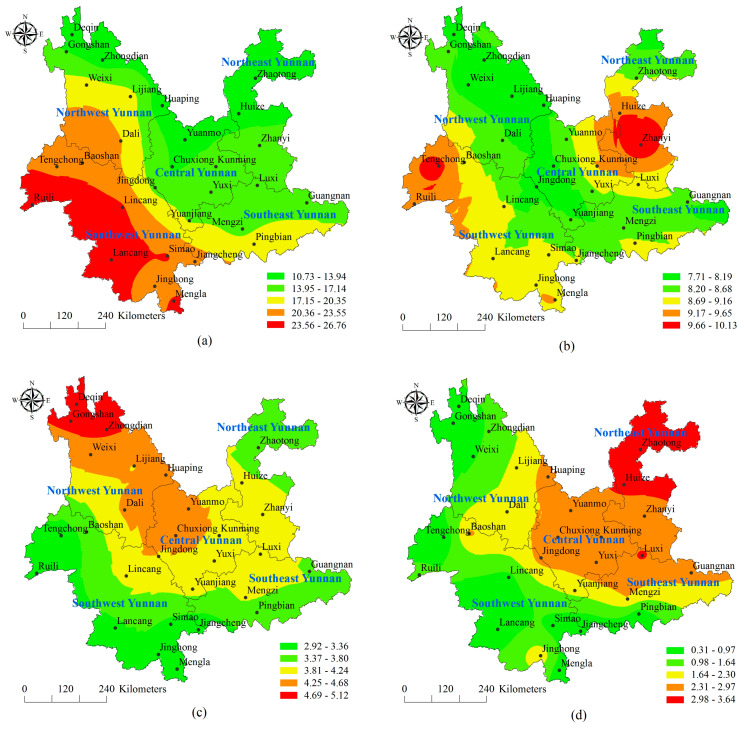
Occurrence frequency distribution of drought event based on different grade in Yunnan from 1960 to 2015 (**a**) slight drought; (**b**) moderate drought; (**c**) severe drought; (**d**) extreme drought.

The suitable drought index for each study area was selected to calculate the drought frequency at each station according to the drought grades from Table 1. The spatial distribution characteristics were shown in Figure 3. From Figure 3a, the slight drought was a zonal increasing distribution from the northeast to the southwest in Yunnan province in 1960–2015 the high value areas were mainly in Lancang and Ruili in the Southwest Yunnan. The frequency is 9.09–27.73% and occurred with an average of once every six years. Figure 3b shows that the high frequency areas of the moderate drought were mainly distributed in Huize and Zhanyi in the Northeast Yunnan, Kunming in the Central Yunnan and some areas in the Southwest Yunnan, with the highest frequency is 16.67%, once in 12 years on average. For the distribution of severe drought (Figure 3c), the frequency of Deqin, Gongshan and Zhongdian was the highest in Northwest Yunnan, then the Dali, Lijiang and Chuxiong and the lowest frequency was in Southwest Yunnan. From Figure 3d, the distribution of the extreme drought was in a decreasing band from northeast to southwest, and its spatial distribution characteristics were just opposite to that of the slight drought. The highest value areas were in Zhaotong and Huize in the Northeast Yunnan, where the occurrence frequency of the slight drought was lowest. The frequency is 0.3–3.64%, which is the lowest in all levels of the drought, once in 94 years on average.

### 4.2. Drought Event Identification

The drought events were identified under different drought index thresholds using trial-and-error method and compared some points of the events with the actual ones including the total number, the occurrence time and the regions. For example, according to the recording in the *China Meteorological Disaster code* (*Yunnan volume*), the spring and summer drought occurred in 1979, and the drought severity was the highest from January to May. The disaster affected areas include 15 regions, among which the Central Yunnan, Zhaotong, Huize and other regions were extreme drought and most of the other regions are moderate or severe drought. Hence, when adjusting the threshold value, it was necessary to be able to accurately identify the drought. Three threshold values of each drought index were determined based on national standard for classification of meteorological drought [32] and previous research [7,48] (Table 4).

### 4.3. Determination of Copula

#### 4.3.1. Determination of the Marginal Distribution of Drought Characteristic Variables

According to Table 1, appropriate drought indexes were selected and Theory of Runs was used to separate the drought duration and severity of the 29 stations. While Kendall’s rank correlation coefficient was calculated. The correlation coefficient is 0.72–0.89 and passed the test of 0.05 significance level. That was showed that the correlation between drought duration and drought severity was high, which can be used copula function to establish the joint distribution function of the two drought characteristic variables.

Six distribution test statistics of drought duration and drought severity were calculated, respectively, i.e., the maximum difference between the empirical cumulative distribution and the theoretical distribution. The distribution function corresponding to the minimum test statistics of drought duration in 21 stations was Weibull distribution in all the 29 stations, accounting for 72.4% of the total number of stations. Therefore, Weibull distribution was selected as the distribution function of the drought duration. The distribution function corresponding to the minimum test statistics of drought severity in 18 stations was Gamma distribution, accounting for 62.1% of the total number of stations. Therefore, the optimal distribution function of the drought severity was Gamma distribution.

K–S test of 29 stations was illustrated with Kunming Station as an example. The sample size of drought events in Kunming station was 66. At the significance level of 0.05, the critical value of K–S test was 0.17, and the statistics of the drought duration and severity were 0.09 and 0.12, respectively, which all were less than the critical value of 0.17. Therefore, under the significance level of 0.05, the drought duration and severity obey Weibull distribution and Gamma distribution, respectively.

#### 4.3.2. Determination of the Joint Distribution Function of the Drought Characteristic Variables

The goodness-of-fit test the joint function was carried out by calculating values of RMSE and AIC between the theoretical copula and the empirical copula. the results were shown in Table 5. Values of RMSE and AIC of Frank copula were minimum in 23 stations, such as Zhaotong, Huize, Zhanyi, Guangnan, that accounting for 79.3% of the total stations. In addition, values of RMSE and AIC of Frank copula was close to the values of the best goodness-of-fit copulas in Luxi, Jingdong, Baoshan Weixi, Zhongdian and Jinghong stations. For example, the RMSE and AIC of Clayton copula in Luxi station are 0.0388 and −257.95, the RMSE and AIC of Frank copula were 0.0485 and −240.10, the difference of RMSE and AIC were only 0.0097 and 17.85. Therefore, Frank copula function is selected as the joint distribution function of drought duration and drought intensity in this study.

### 4.4. Joint Probability

**Figure 4:**
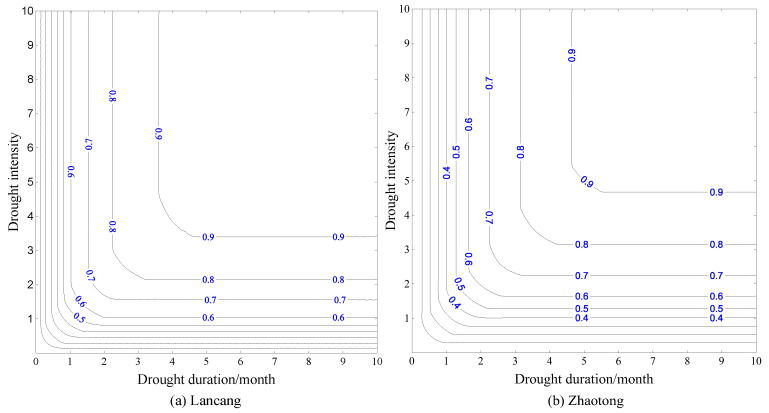
Joint cumulative probability contour line (**a**) Lancang; (**b**) Zhaotong.

The joint probability of drought duration and drought severity, that means the probability of occurrence of drought events when both drought duration and drought severity were less than or equal to a given value, were calculated used by Equation (3). According to the analysis of the Section 4.1, the probability distribution of Zhaotong station with the highest frequency of extreme drought (Northeast Yunnan) and Lancang station with the highest frequency of slight drought (southwest Yunnan) were selected as an example (Figure 4).

From Figure 4, the drought events were mostly short-term drought duration and high drought severity, long-term drought duration and low drought severity at Lancang and Zhaotong stations. The joint cumulative probability of the two stations increased with the increase of the drought duration and severity. The density of the joint probability isoline corresponding to the drought duration and severity of Lancang station was higher than that of Zhaotong station, which indicated that the drought events of short-term drought duration and low drought severity in the region occurred frequently at Lancang station. The events with drought duration less than 1 month, and the drought severity less than 1 account for about 45% of all the drought events; while events with drought duration less than 4 months, and drought severity less than 4 account for about 90% of all the drought events; the events with drought duration more than 6 months, and drought severity more than 4 rarely take place. The events in Zhaotong station with drought duration less than 1 month, and drought severity less than 1 accounts for about 25% of all drought events, which was less than that of Lancang station; the events with drought duration more than 6 months, and drought severity more than 4 were more than those of Lancang station. The conclusion in 4.1 of the study was further verified that the slight drought mostly occurred in the Southwest Yunnan, while the extreme drought mainly occurred in the Northeast Yunnan.

There were many drought events with high severity in Yunnan Province during 2009–2013. A rare drought event occurred with the severity of 2.76 from January 2010 to August 2010 at Lancang station. From Figure 4a, that the joint probability of drought event reached this severity was about 0.85. The most serious drought event with the severity of 9.85, lasting for ten months, occurred from March 2011 to December 2011 at Zhaotong station, which was an exceedingly rare drought event in history. In addition, the joint probability distribution results of other stations are similar to the Lancang station and Zhaotong station.

### 4.5. Joint Return Period

**Figure 5:**
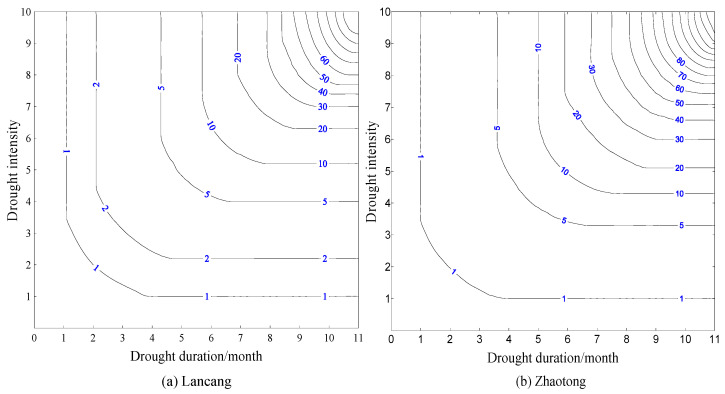
Joint return period contour line (**a**) Lancang; (**b**) Zhaotong.

The drought of severity (duration) was considered as returned once, when the value of the drought severity (duration) was greater than a given one. Taking the stations of Lancang and Zhaotong as examples in this study. From Figure 5, the joint return period of the two stations showed an increasing trend with the increase of the drought duration and drought severity. The return periods of the drought with short duration and high severity and long duration and low severity were short. The drought events with short duration and high severity such as 2 and 10, respectively, the return periods of these events were 1.9 months and 2.0 months at Lancang station and Zhaotong station, respectively.

The isoline of return period of Lancang station was sparse compared with Zhaotong station, especially for the drought events with long duration and high severity, which indicated that the frequency of the drought events in the region where Lancang station was located was low. Furthermore, that verified the frequency of the drought events in the Northeast Yunnan was higher than that in the Southwest Yunnan.

For the drought events with the same return period, such as 80 a, the drought severity of Lancang station was 8.7, and the drought duration was 9.9 months. The corresponding drought severity of Zhaotong station at that time was 8.0, lasting for 9.2 months. That showed such drought events were more likely to occur in Zhaotong station than that in Lancang station. In addition, the joint return period distribution results of other stations are similar to the Lancang station and Zhaotong station.

### 4.6. Application of Joint Return Period in Agrometeorological Disasters

In the study, the Zhaotong station and Lancang station were taken as examples. From Table 3, there were 11 drought events with drought duration less than 4 months, and drought severity less than 4 in Zhaotong station area during 1991–2015, and the return period of these drought events was in the range of 0.9–4.5 a. With the increase of the drought duration and severity, the frequency of the drought events was smaller and the return period was longer. The years of the longer joint return period were 2006, 2010, 2011 and 2012. According to the data of China Planting Information Network, the drought was most serious in 2006, 2010, 2011 and 2012, with the output decreased by 139,000, 165,000, 288,000 and 173,000 hm^2^, respectively. There were 22 drought events of the drought duration less than four months and the drought severity less than four in the area where Lancang station was located. The return period of these drought events was in the range of 0.9–3.5 a. With the increase of drought duration and severity, the frequency of drought events is smaller and the joint return period is longer. The years of the longer joint return period were 2009, 2010 and 2011. According to the data of China Planting Information Network, the drought was most serious in 2009, 2010 and 2011, with the production reduced by 9800, 13,400 and 18,800 hm^2^, respectively. Therefore, it can be concluded that the joint return period of drought duration and drought severity can better reflect the year of agricultural disaster in Yunnan Province. The larger the joint return period is, the larger the area of agricultural disaster is. Furthermore, the correct estimation of the return period of different drought events can provide more scientific guidance for the prevention and treatment of the agrometeorological drought.

The drought characteristic analysis of the theoretical model in this study was verified, based on the ten-day data sets (China meteorological data sharing service system) of Zhaotong and Lancang stations from 1991 to 2015 and the data obtained from the two-dimensional copulas. The results (Table 6 and Table 7) showed that the actual drought duration and the corresponding theoretical drought duration were almost the same, with an error of 0.1–0.4 a, for the drought events of the same drought level. While the drought characteristics extracted from the drought event by the Theory of Runs were consistent with the actual drought characteristics when comparing the theoretical return period and the actual return period of the same drought event. The simulated return period can better reflect the actual drought situation, that further indicated the combination of Theory of Runs and copula can better describe the drought characteristics of Yunnan Province. Furthermore, the results will guide agricultural production and prevent and control drought events occur.

## 5. Discussion

The characteristics of drought in Yunnan Province were studied from three aspects including the spatial characteristics of drought frequency, the joint cumulative probability and the joint return period. The results showed that the Northeast Yunnan was the high-risk area of drought and the Southwest Yunnan was the low risk area of drought. These results were consistent with the research by Chang Wenjuan et al. [49], who studied the drought risk of Yunnan used by Principal Component Analysis. Furthermore, it also showed that the Theory of Runs and the Copula in the study were reliable for the research of drought in Yunnan Province.

Both the selection of drought index and the identification of drought index threshold value are especially important in the analysis of drought events. There is no uniform rule for the selection of threshold based on the Theory of Runs. The applicability of each drought index is different-in-different regions. Currently, the method of setting only a single drought index and one truncation level was mostly used to identify drought events [50,51,52], which could easily reduce the accuracy of drought identification. In the study, four drought indexes have been used for different seasons and regions in Yunnan, three threshold values of each drought index were set by the trial-and-error method, which may improve the reliability and rationality in drought identification [7,48,53,54].

Currently, there are many studies on the simulation of return period of drought events by the copula function [55,56,57]. However, those studied were not applied the agricultural disaster. Based on the analysis of the return period, this study applied the results of the simulation to the actual agricultural disaster, and the combined return period could accurately reflect the year of agricultural disaster in Yunnan Province. These results could also be used to the drought prevention. However, due to the different types of dry crops and the different water demand in each growth period, the disaster degree of crops is related to the growth period of crops and the occurrence of meteorological drought events. The coupling of crop water deficit and agricultural drought needs further study.

To date, there are hundreds of copula functions available, but only a few are mature in practical application. In this study, only four copula functions were discussed, and other copula types would be the focus of future research [43,58]. In addition, this study established two-dimensional joint distribution model of drought duration and severity. Some researchers used three or four characteristic variables, which could reflect drought characteristics better [59,60]. However, with the increase of the number of drought characteristic variables, the difficulty of parameter estimation and calculation will increase accordingly. Hence, the solution of copula function based on multiple characteristic variables, and the application of high-dimensional copula will become a new direction in future research.

The results of this study can provide a reference for the prediction, assessment, agricultural management and decision making on drought in Yunnan Province. Definitely, the good performance of the finding in Yunnan Province does not mean it could work in any other places. However, we believe the methodology could be extended to other areas over the world. Furthermore, for the regions with similar topographical and climate conditions, our work could be a reference for the application of drought.

## 6. Conclusions

Based on the Theory of Runs, the drought events of the drought duration and severity were abstracted in Yunnan Province from 1960 to 2015. The joint distribution function of these two drought characteristic variables was established by copulas. The joint cumulative probability and joint return period between the two characteristic variables were analyzed, and the relationship between that and the actual crop drought reduction area were also studied. The following conclusions were drawn from the results.

(1) The area of the slight drought was zonal increasing distribution from the northeast to the southwest in Yunnan province in 1960–2015; the areas of the high frequency moderate drought were mainly distributed in Huize and Zhanyi in the Northeast Yunnan, Kunming in the Central Yunnan and some areas in the Southwest Yunnan; the areas of the high frequency severe drought were mainly distributed in Deqin, Gongshan and Zhongdian in Northwest Yunnan; the distribution of the extreme drought was a decreasing band from northeast to southwest;

(2) The marginal distribution function of the drought duration was Weibull distribution, and the drought severity was Gamma distribution in Yunnan Province. The Frank copula, which was the most fitting function, was used to build the joint distribution function in order to analyze the joint cumulative probability of the drought characteristic variables and the joint return period of the drought events. The joint cumulative probability and the joint return period would increase with the increase of the drought duration and severity;

(3) Based on the combined analysis of the return period of drought events and the actual drought area, the characteristics of the drought events were almost consistent to the actual drought characteristics, and the error range between the theoretical return period and the actual was 0.1–0.4 a. The year of the agricultural disaster can be accurately reflected by the combined return period in Yunnan Province. The research can provide guidelines for the agricultural management in the drought area.

## Figures and Tables

**Table 1:** Suitable drought index in different regions and seasons.

Region.	Winter	Spring	Summer	Autumn
Northwest Yunnan	SPI	SPI	SPI	SPI
Southwest Yunnan	Pa	Pa	SPEI	SPEI
Central Yunnan	SPI/Pa	SPI/Pa	SPEI	SPEI
Northeast Yunnan	SPI	SPI	SPEI	SPEI
Southeast Yunnan	CI_new_	CI_new_	SPEI	SPEI

Note: SPI is standardized precipitation index; SPEI is standardized precipitation evapotranspiration index; Pa is percentage of precipitation anomaly; CI_new_ is improved comprehensive meteorological drought index.

**Table 2:** Drought grades based on SPI, SPEI, Pa and CI_new_ values.

SPI	SPEI	Pa	CI_new_	Drought Category
−0.5 < SPI	−0.5 < SPEI	−0.4 < Pa	−0.6 < CI_new_	No drought
−1.0 < SPI ≤ −0.5	−1.0 < SPEI ≤ −0.5	−0.6 < Pa ≤ −0.4	−1.2 < CI_new_ ≤ −0.6	Slight drought
−1.5 < SPI ≤ −1.0	−1.5 < SPEI ≤ −1.0	−0.8 < Pa ≤ −0.6	−1.8 < CI_new_ ≤ −1.2	Moderate drought
−2.0 < SPI ≤ −1.5	−2.0 < SPEI ≤ −1.5	−0.95 < Pa ≤ −0.8	−2.4 < CI_new_ ≤ −1.8	Severe drought
SPI ≤ −2.0	SPEI ≤ −2.0	Pa ≤ −0.95	CI_new_ ≤ −2.4	Extreme drought

Note: From national standard for classification of meteorological drought [32].

**Table 3:** Archimedean copula functions and the parameters estimation.

Copula Type	Copula Formula	Relationship between θ and τ
Gumbel–Hougaard	$C\left(u,v\right)=\exp\left\{-{\left[{\left(-\ln u\right)}^{\theta }+{\left(-\ln v\right)}^{\theta }\right]}^{1/\theta }\right\}$	$\tau =1-\theta^{-1},\theta \geq 1$
Ali–Mikhail–Haq	C(u,v)=uv/[1−θ(1−u)(1−v)]	$\tau =\left(1-\frac{2}{3\theta }\right)-\frac{2}{3}{\left(1-\theta^{-1}\right)}^{2}\ln\left(1-\theta \right),-1\leq \theta \leq 1$
Frank	$C\left(u,v\right)=-\frac{1}{\theta }\ln\left[1+\frac{\left(e^{-\theta u}-1\right)\left(e^{-\theta v}-1\right)}{e^{-\theta }-1}\right]$	$\tau =1+\frac{4}{\theta }\left(\frac{1}{\theta }\int_{0}^{\theta }\frac{t}{e^{t}-1}dt-1\right),\theta \in R$
Clayton	$C\left(u,v\right)={\left(u^{-\theta }+v^{-\theta }-1\right)}^{-1/\theta }$	$\tau =\frac{\theta }{\theta +2},\theta \geq 0$

Note: *u* and *v* represent dependent cumulative distribution functions of univariate distributions of random variables.

**Table 4:** Drought identification threshold of the SPI, SPEI, Pa and CI_new_.

Drought Index	Rainy Season (May-September)			Non-Rainy Season (October-April)		
	R_0_	R_1_	R_2_	R_0_	R_1_	R_2_
SPI	0	−0.5	−1.0	0	−0.5	−1.0
SPEI	0	−0.5	−1.0	0	−0.5	−1.0
Pa	0	−0.4	−0.6	0.2	−0.4	−0.6
CI_new_	0	−0.6	−1.2	0.2	−0.6	−1.2

**Table 5:** Goodness-of-fit tests for copula.

Station Number	Station	Gumbel–Hougaard		Ali–Mikhail–Haq		Frank		Clayton	
		RMSE	AIC	RMSE	AIC	RMSE	AIC	RMSE	AIC
1	Zhaotong	0.0553	−356.98	0.1132	−268.15	0.0414 *	−392.87 *	0.0461	−379.54
2	Huize	0.0564	−302.78	0.1255	−218.00	0.0322 *	−362.19 *	0.0528	−309.77
3	Zhanyi	0.0678	−310.18	0.0994	−265.80	0.0461 *	−354.93 *	0.0652	−314.71
4	Luxi	0.0673	−213.89	0.1046	−178.61	0.0485	−240.10	0.0388 *	−257.95 *
5	Guangnan	0.0467	−304.40	0.1284	−203.26	0.0374 *	−326.61 *	0.0402	−319.39
6	Pingbian	0.0598	−437.41	0.1325	−313.30	0.0527 *	−457.13 *	0.0566	−445.99
7	Mengzi	0.0632	−274.15	0.1068	−221.68	0.0498 *	−297.97 *	0.0549	−288.22
8	Deqing	0.0595	−235.03	0.0984	−192.77	0.0459 *	−256.83 *	0.0482	−252.72
9	Gongshan	0.0641	−437.57	0.1302	−324.19	0.0546 *	−463.24 *	0.0674	−429.54
10	Weixi	0.0214 *	−267.11 *	0.0867	−169.17	0.0392	−224.74	0.0413	−221.08
11	Zhongdian	0.0269 *	−301.71 *	0.1162	−178.81	0.0447	−259.05	0.0658	−226.58
12	Lijiang	0.0562	−320.43	0.1008	−255.00	0.0432 *	−349.89 *	0.0443	−347.08
13	Dali	0.0438	−335.84	0.0925	−255.10	0.0361 *	−356.72 *	0.0475	−327.08
14	Huaping	0.0602	−279.01	0.1226	−207.88	0.0335 *	−337.62 *	0.0458	−306.35
15	Baoshan	0.0534	−261.70	0.1347	−178.42	0.0387	−290.67	0.0209 *	−346.12 *
16	Tengchong	0.0722	−444.81	0.1389	−333.58	0.0545 *	−492.62 *	0.0657	−460.85
17	Ruili	0.0599	−335.81	0.1231	−249.37	0.0429 *	−375.87 *	0.0463	−366.71
18	Lincang	0.0569	−313.31	0.1334	−219.58	0.0343 *	−368.99 *	0.0422	−346.19
19	Lancang	0.0435	−399.28	0.1273	−261.83	0.0297 *	−448.13 *	0.0348	−427.84
20	Jingdong	0.0545	−288.96	0.1089	−219.73	0.0388	−322.93	0.0306 *	−346.68 *
21	Simao	0.0474	−382.19	0.1332	−252.00	0.0354 *	−418.97 *	0.0440	−391.57
22	Jinghong	0.0687	−260.44	0.0347*	−327.38 *	0.0402	−312.96	0.0459	−299.97
23	Mengla	0.0546	−381.82	0.1077	−292.15	0.0467 *	−402.45 *	0.0485	−397.46
24	Jiangcheng	0.0695	−451.29	0.1115	−370.93	0.0532 *	−496.73 *	0.0646	−463.72
25	Yuanjiang	0.0663	−339.91	0.1369	−248.55	0.0449 *	−389.02 *	0.0487	−378.78
26	Yuanmou	0.0416	−360.48	0.1032	−256.90	0.0298 *	−398.51 *	0.0371	−373.53
27	Kunming	0.0628	−330.14	0.1141	−258.48	0.0379 *	−390.74 *	0.0449	−370.40
28	Chuxiong	0.0582	−327.89	0.1273	−237.10	0.0485 *	−349.04 *	0.0555	−333.40
29	Yuxi	0.0605	−300.95	0.1204	−226.63	0.0392 *	−347.82 *	0.0424	−339.35

Note: * represents the minimum RMSE and AIC values for the corresponding station.

**Table 6:** Comparison of drought characteristics between theoretical and actual drought events in Zhaotong from 1991 to 2015.

Year	Drought Duration		Return Period (Years)		Drought Grade	Crop Production Area (Million hm^2^)
	Actual	Theoretical	Actual	Theoretical		
1991	03−06	03−06	1.5	1.7	Slight drought	1.2
1992	06−11	06−10	6.7	6.6	Moderate drought	4.6
1993	06−12	06−12	10.8	10.5	Severe drought	10.2
1994	04−06	04−07	2.6	2.6	Slight drought	2.5
1995	02−04	02−05	1.5	1.2	Slight drought	2.4
1996	03−05	03−06	1.0	0.9	Slight drought	3.6
1997	03−05	03−05	0.9	0.8	Slight drought	3.9
1998	02−07	03−07	4.2	4.3	Moderate drought	6.7
1999	02−05	02−06	2.6	2.2	Slight drought	4.2
2000	08−11	08−10	1.8	1.6	Slight drought	3.9
2001	03−08	03−08	5.0	5.3	Moderate drought	7.6
2002	04−10	04−10	9.6	9.8	Moderate drought	8.1
2003	01−10	02−10	19.5	19.3	Severe drought	12.4
2004	08−11	08−11	4.4	4.2	Moderate drought	7.8
2005	03−09	03−09	13.8	13.4	Severe drought	11.3
2006	02−08	02−09	12.0	11.7	Severe drought	13.9
2007	02−05	02−06	2.1	2.2	Slight drought	4.1
2008	03−08	04−08	4.3	4.5	Moderate drought	8.5
2009	06−12	06−12	9.8	9.8	Moderate drought	8.5
2010	01−09	02−09	25.5	25.5	Extreme drought	16.5
2011	03−12	02−12	108.2	108.5	Extreme drought	28.8
2012	03−10	03−10	22.3	22.7	Extreme drought	17.3
2013	02−08	02−09	17.8	17.5	Severe drought	12.6
2014	04−07	03−07	4.5	4.3	Moderate drought	6.6
2015	05−08	04−08	3.9	3.8	Moderate drought	5.9

**Table 7:** Comparison of drought characteristics between theoretical and actual drought events in Lancang from1991 to 2015.

Year	Drought Duration		Return Period (Years)		Drought Grade	Crop Production Area (Million hm^2^)
	Actual	Theoretical	Actual	Theoretical		
1991	03−03	03−04	0.8	0.8	Slight drought	0.13
1992	05−08	05−09	3.5	3.6	Moderate drought	0.45
1993	07−09	07−09	2.5	2.7	Slight drought	0.22
1994	04−04	04−04	0.7	0.9	Slight drought	0.18
1995	03−04	03−04	1.2	1.5	Slight drought	0.25
1996	03−03	03−04	0.5	0.5	Slight drought	0.12
1997	05−06	05−06	1.0	0.8	Slight drought	0.23
1998	03−05	03−06	4.0	4.2	Moderate drought	0.65
1999	02−03	02−02	1.8	1.8	Slight drought	0.29
2000	04−04	03−04	0.7	0.7	Slight drought	0.14
2001	03−05	03−06	1.6	1.5	Slight drought	0.20
2002	03−05	03−05	1.3	1.4	Slight drought	0.21
2003	04−06	04−06	2.0	1.7	Slight drought	0.27
2004	03−04	03−04	1.3	1.3	Slight drought	0.19
2005	04−06	04−07	2.5	2.7	Slight drought	0.33
2006	06−07	06−08	0.9	0.9	Slight drought	0.18
2007	06−06	06−07	0.8	0.8	Slight drought	0.18
2008	05−07	05−07	1.6	1.4	Slight drought	0.37
2009	07−12	07−11	7.6	7.5	Severe drought	0.98
2010	01−08	01−09	31	31.4	Extreme drought	1.88
2011	04−09	05−09	10.2	10.2	Severe drought	1.34
2012	01−04	01−05	3.4	3.2	Moderate drought	0.51
2013	03−06	02−06	3.5	3.6	Moderate drought	0.56
2014	05−08	05−09	1.9	1.9	Slight drought	0.29
2015	06−08	06−08	1.3	1.2	Slight drought	0.22
